# Soluble hexamethyl-substituted subphthalocyanine as a dopant-free hole transport material for planar perovskite solar cells

**DOI:** 10.1098/rsos.180617

**Published:** 2018-08-01

**Authors:** Feng Wang, Xiaoyuan Liu, Ehsan Rezaee, Haiquan Shan, Yuxia Zhou, Zong-Xiang Xu

**Affiliations:** Department of Chemistry, South University of Science and Technology of China, 518055 Shenzhen, Guangdong, People's Republic of China

**Keywords:** planar perovskite solar cell, hexamethyl-substituted subphthalocyanine, dopant-free hole transport material

## Abstract

Boron subphthalocyanine (SubPc) has special physical and chemical properties, originating from its non-centrosymmetric, near-planar taper structure and large conjugated system; it can act as an alternative to the small molecule hole-transporting material 2,2′,7,7′-tetrakis-(*N*,*N*-di-*p*-methoxyphenylamine)-9,9′-spirobifluorene in perovskite solar cells (PSCs). To achieve a higher solubility in common organic solvents and a more suitable highest occupied molecular orbital energy level that aligns with the valence band of the perovskite material, a SubPc molecule with a hexamethyl substitution at its peripheral position (Me_6_-SubPc) was successfully designed and synthesized in a one-step method. Completely solution processed PSCs were fabricated with only a small hysteresis, a power conversion efficiency of 6.96% and *V*_oc_ of 0.986 V.

## 1. Introduction

Owing to their low cost, high open circuit voltage and simple structure, organic–inorganic lead halide perovskite solar cells (PSCs) have attracted significant attention since first being reported by the Miyasaka group. The power conversion efficiency (PCE) of PSCs has increased from 3.8% in 2009 to 22.1% in 2016 [1–5].

These highly efficient PSCs always use light-absorbing sensitizers, electron transporting materials (ETMs) and hole-transporting materials (HTMs) [6–8]. HTMs serve as hole transport channels, which facilitates hole extraction and retards charge recombination at the interfaces between the HTMs and the perovskite layer and between the HTMs and the ETMs [9,10]. Ideally, a HTM should have the following characteristics: excellent hole transport capacity and conductivity, high mobility, good organic solubility and low cost; further, the highest occupied molecular orbital (HOMO) energy level should be well aligned with the valence band of the perovskite material. The state-of-the-art commercial HTM 2,2′,7,7′-tetrakis-(*N*,*N*-di-*p*-methoxyphenylamine)-9,9′-spirobifluorene has been widely used in PSCs [11–13]. However, its complex synthesis steps limit its commercial applications. It also has an inherently low hole mobility [14]. Doping the material with lithium bis(trifluoromethanesulfonyl)imide and 4-*tert*-butyl pyridine can improve its hole mobility so that PSCs with impressive PCE values can be fabricated. However, because bis(trifluoromethanesulfonyl)imide and 4-*tert*-butyl pyridine are deliquescent and have poor thermal stabilities, doping very negatively affects the stability of PSCs [15–20]. Therefore, researchers worldwide are committed to developing inexpensive and efficient dopant-free HTMs, such as inorganic HTMs, polymer HTMs and small-organic HTMs [9,16,19,21–26].

Recently, the organic small molecule phthalocyanine (Pc) has been successfully applied to PSCs as a HTM because of its simple synthesis route, high thermal and chemical stability, high hole mobility and long exciton diffusion length [27–32]. Professor Lianos of the University of Patras, Greece, first reported the use of unmodified copper phthalocyanine (CuPc) as a hole-transporting layer in a PSC with a PCE of 5% [27]. Professor Nazeeruddin's research team prepared HT-ZnPc by modifying 5-hexylthiophene on zinc phthalocyanine and fabricated PSCs via a liquid phase process without doping, reaching a maximum fill factor (FF) of 80.3% and a PCE of 17.1% [28]. Our group reported PCEs of 8.5% and 16.28% for soluble tetrabutyl copper phthalocyanine and insoluble octamethyl palladium phthalocyanine HTM, respectively [29,30]. However, it should be mentioned that unmodified metal phthalocyanines have poor solubilities and that the synthesis of soluble phthalocyanines is both complicated and costly.

Boron subphthalocyanines (SubPcs) have similar physico-chemical properties to phthalocyanine: a delocalized 14π electron system, good chemical and thermal stability, one-step synthesis route, simple purification, conducive to industrialization. SubPcs also have high modification and can obtain different optoelectronic properties by modifying different groups in the α, β position or on the central B atom to meet the needs of different optoelectronic devices [33–41]. However, SubPc also has a poor organic solubility (similar to Pc) and its HOMO level is too low to align with the valence band of the perovskite material, which limits its potential application in PSCs [42].

In this paper, we report on the synthesis of hexamethyl-substituted subphthalocyanine (Me_6_-SubPc) using a one-step method. The modification of methylhexamethyl at the peripheral position affords the material a good organic solubility and changes the HOMO level from −5.6 eV (SubPc) to −5.3 eV for Me_6_-SubPc. PSCs with Me_6_-SubPc as a HTM were prepared using a completely liquid phase process. These solar cells had a non-optimized PCE of 6.96% and a *V*_oc_ of 0.986 V, demonstrating that further optimization may significantly improve device performances.

## 2. Experimental sections

### 2.1. Materials

Unless specified otherwise, all materials were purchased and used as received. [6,6]-phenyl-C61-butyric acid methyl ester (PCBM) and organic solvents were purchased from Sigma Aldrich. 4,5-dimethylphthalonitrile was purchased from Shanghai Run Biotech. CH_3_NH_3_I and PbI_2_ were purchased from TCI. Fluoride-doped tin oxide (FTO) glass slides with a sheet resistance of 14 ohm square^−1^ were purchased from Pilkington.

### 2.2. Hexamethyl-substituted subphthalocyanine (Me_6_-SubPc) synthesis

Me_6_-SubPc was synthesized with a modified version of a method reported in the literature [35,36]. A suspension of 4,5-dimethylphthalonitrile (3.12 g, 0.02 mol) and dichlorobenzene (30 ml) was stirred at room temperature under Ar. Then, boron trichloride (20 ml, 0.02 mol, 1 M in dichloromethane (DCM)) was added through a cannula into the suspension. Then, the reaction mixture was heated to 600°C using a heat gun while being stirred to remove the DCM; then the mixture was heated for 5 min. After the reaction concluded, the mixture was cooled to room temperature. The product was purified by column chromatography on silica gel using petroleum ether to remove dichlorobenzene and petroleum ether–ethyl acetate (3 : 1) as eluent. The solvent was removed by rotary evaporation and a purple powder was obtained. A single crystal was obtained from DCM methylene chloride and petroleum ether at low temperature. The ^1^H-nuclear magnetic resonance (^1^H-NMR) results are as follows. ^1^H-NMR (400 MHz, CDCl_3_): *δ* = 7.46–7.35 (6H, d), 2.33–2.17 (18H, d). Electrospray ionization-mass spectrometry (ESI-MS): *m/z* (M + H)^+^ = 515.19. Ultraviolet–visible (UV–Vis) (CH_2_Cl_2_): *λ*_max_ = 572 nm. Infrared (potassium bromide): *ν* = 3422, 3048, 2968, 2920, 2440, 1765, 1717, 1622, 1553, 1464, 1375, 1294, 1227, 1142, 1020, 974, 876, 793, 708, 623 cm^−1^.

### 2.3. Characterization of hexamethyl-substituted subphthalocyanine

^1^H-NMR spectra were obtained from a CDCl_3_ solution on a Bruker Ascend 400 NMR spectrometer. High-resolution mass spectrometry was recorded on a Q-Exactive mass spectrometer. The UV–Vis spectrum of the solution (in CH_2_Cl_2_) was recorded on a PerkinElmer Lambda750S spectrophotometer. The HOMO level was measured directly using an ionization energy measurement system (IPS-4).

### 2.4. Fabrication and characterization of perovskite solar cells

The structure of the PSCs fabricated in this study was FTO/SnO_2_/PCBM/perovskite/Me_6_-SubPc/Au on patterned FTO glass. The patterned FTO glass was washed with detergent, ethanol, chlorobenzene and acetone in an ultrasonic bath, subsequently dried with nitrogen stream, and finally treated with ultraviolet-ozone for 20 min. A SnO_2_ electron transport layer (ETL) was prepared according to a method we reported on previously [28]. A 0.1 M precursor solution of SnO_2_·2H_2_O in ethanol was spin coated on the surface of the cleaned FTO substrates with a spin speed of 2000 revolutions per minute (r.p.m.) for 45 s to obtain a compact and uniform SnO_2_ ETL. Then, the FTO substrates coated with the SnO_2_ ETL were annealed on a hotplate in air at 180°C for 1 h; 15 mg PCBM was dissolved in 1 ml chlorobenzene, and the solution was spin coated onto the surface of the FTO /SnO_2_ ETL samples with a spin speed of 2000 r.p.m. and heated on a hotplate at 100°C for 10 min. The perovskite layer (thickness of about 500 nm) was prepared by solvent engineering. The thickness of the perovskite layer was gauged using an Ambios Technology (Santa Cruz, USA) XP-2 profilometer. The Me_6_-SubPc solutions were prepared by dissolving 15 mg Me_6_-SubPc in 1 ml chlorobenzene. The Me_6_-SubPc solution was spin coated onto the surface of the perovskite layer with a spin speed of 4000 r.p.m. for 30 s. The Au electrodes were subsequently deposited through thermal evaporation under a vacuum of approximately 1 × 10^−6^ T; the electrode thickness was monitored *in situ* using quartz crystal monitors during the evaporation.

The current density–voltage (J–V) curves were measured with a computer-controlled Keithley 236 source measurement unit coupled with a Zolix ss150 solar simulator. A xenon lamp coupled with AM1.5 solar spectrum filters was used as the light source, and the optical power at the sample was 100 mW cm^−2^. The intensity of the solar simulator's light was calibrated using a standard silicon solar cell. The external quantum efficiency spectrum was measured using a solar cell quantum efficiency/external quantum efficiency measurement system (Zolix Solar cell scan 100) with a model SR830 DSP lock-in amplifier coupled with a WDG3 monochromator and a 500 W xenon lamp. Thin film stacks with SnO_2_/PCBM/perovskite and SnO_2_/PCBM/perovskite/Me_6_-SubPc structures on FTO patterned glass were fabricated following the same procedure as for PSC fabrication. Grazing incidence X-ray diffraction patterns of the samples were recorded on a Smartlab 9 kW diffractometer with a Göbel mirror attachment. The irradiation occurred with a parallel CuK*_α_*_1,2_ X-ray beam (grazing incident angle of 2.000° (*θ*)); the detector was set to collect the diffraction data in a 2*θ* range of 3–30° with a step-size of 0.02° (2*θ*) at a fixed speed of 1 s step^−1^. The UV–Vis spectrum of a Me_6_-SubPc film was recorded on a PerkinElmer Lambda750S spectrophotometer. The Me_6_-SubPc film was prepared by spin-coating a solution of 15 mg Me_6_-SubPc in chlorobenzene onto a cleaned FTO substrate. The morphologies of the thin film samples were probed using atomic force microscopy (AFM) with a Keysight Technologies (5500AFM/STM) scanning probe in tapping mode. Steady-state photoluminescence (PL) spectra were measured by FLS980 Spectrometer-Edinburgh Instruments. The wavelength of excitation light was 470 nm and the samples were excited from the perovskite or Me_6_-SubPc layer. Impedance spectroscopy measurements were carried out by CHI 760E under illumination conditions with different direct current bias potentials from 0.3 to 0.8 V and frequency from 100 kHz to 0.01 Hz. The carrier mobility was extracted by fitting the J–V curves according to the modified Mott–Gurney equation [43]:

2.1 $$J=\frac{9}{8}\varepsilon_{0}\varepsilon_{r}\mu \frac{V^{2}}{d^{3}},$$

where *J* is the current density, *ϵ*_0_ is the permittivity of free space, *ϵ*_r_ is the relative permittivity, *μ* is the zero-field mobility, *V* is the applied voltage and *d* is the thickness of active layer.

## 3. Results and discussion

### 3.1. Synthesis and characterization of hexamethyl-substituted subphthalocyanine

**Scheme 1.**
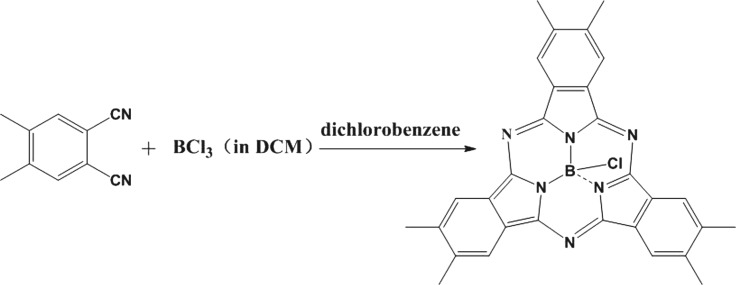
Synthetic route of Me_6_-SubPc.

Me_6_-SubPc was synthesized using a modified version of a previously reported one-step method (synthetic route used in this study shown in scheme 1). The product was recrystallized three times for application in PSCs. The X-ray data for Me_6_-SubPc was collected on a Bruker ApexII diffractometer equipped with a charge-coupled device-area detector using graphite-monochromated Cu K*α* radiation (*k* = 1.54178 Å) generated from a sealed tube source. Data were collected and reduced using the Smart and Saint software [44] in the Bruker package. The structure was solved by means of direct methods using SHELXT-2014 [45,46] and refined via full-matrix least-squares methods based on Fo [45,46] with SHELXL-2014 in the framework of OLEX [47] software. For disordered solvent molecules, as observed on the Fourier density map, a standard Squeeze protocol implemented into Platon software was used to account for areas of unassigned electron density [48]. The non-H atoms found in the electron density map were refined anisotropically. All non-H atoms were placed in calculated positions with the ‘riding-model technique' with displacement parameters bonded to a parent atom. A summary of the crystallographic data and of the refinement parameters for Me_6_-SubPc is provided in table 1.

**Table 1.** Me_6_-SubPc crystallographic data and data collection details.

identification code	er007(4)
empirical formula	C_31_H_28_BN_6_Cl_3_O_2_
formula weight	633.75
temperature (K)	120.03
crystal system	tetragonal
space group	*I*4/*m*
*a* (Å)	18.5385(5)
*b* (Å)	18.5385(5)
*c* (Å)	19.4321(13)
*α* (°)	90
*β* (°)	90
*γ* (°)	90
volume (Å^3^)	6678.3(6)
*Z*	8
*ρ*_calc_ (g cm^−3^)	1.261
*μ* (mm^−1^)	2.781
F(000)	2624.0
crystal size (mm^−3^)	0.4 × 0.2 × 0.2
radiation	CuK*α* (*λ* = 1.54178)
2*Θ* range for data collection (°)	6.59–136.744
index ranges	−22 ≤ *h* ≤ 22, −20 ≤ *k* ≤ 22, −23 ≤ *l* ≤ 21
reflections collected	32 893
independent reflections	3167 [*R*_int_ = 0.1546, *R*_sigma_ = 0.0495]
data/restraints/parameters	3167/0/208
goodness of fit on *F*^2^	1.059
final *R* indexes [*I* >= 2*σ* (*I*)]	*R*_1_ = 0.0826, *wR*_2_ = 0.2329
final *R* indexes (all data)	*R*_1_ = 0.0863, *wR*_2_ = 0.2371
largest diff. peak/hole/eÅ^−3^	1.97/−1.02

**Figure 1.**
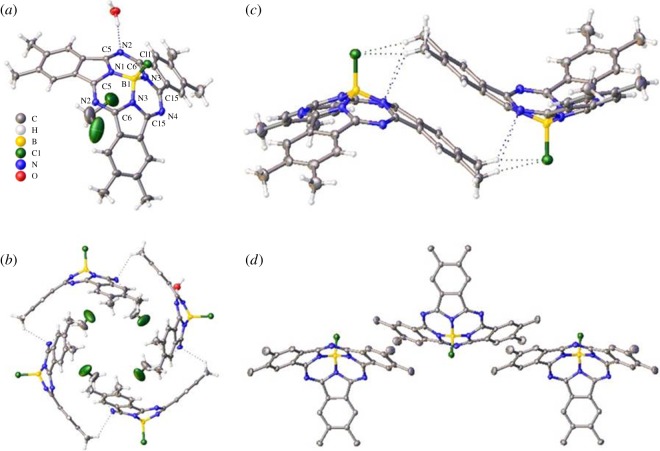
(*a*) ORTEP (Oak Ridge thermal-ellipsoid plot programme) view of Me_6_-SubPc. (*b*) A sphere-like structure containing four Me_6_-SubPc and four CH_2_Cl_2_ molecules. (*c*) Face-to-face interaction π–π stacking. (*d*) Offset interaction π–π stacking.

A perspective view of the Me_6_-SubPc molecule with accompanying atomic labelling scheme is shown in figure 1*a*. Selected bond lengths and angles are presented in table 2. The compound crystallized in the space group *I*4/*m*. In the taper structure of Me_6_-SubPc, a cupped geometry of the delocalized ring system can be observed; it is forced by the tetrahedral boron centre. In this molecule, the boron atom is located nearly 0.6 Å out of the plane of its three bonded nitrogen atoms. The thus created cavity of each molecule is occupied by one CH_2_Cl_2_ molecule and its neighbour Me_6_-SubPc molecules. Sphere-like structures are formed via the contribution of four Me_6_-SubPc molecules along with four CH_2_Cl_2_ molecules with the help of weak CH-N hydrogen bonding interactions (figure 1*b*). The packing arrangements of the crystal lattice result from π–π stacking interactions between sphere-like structures, as well as weak hydrogen bonding. As shown in figure 1*c*,*d*, two different π–π stacking interactions can be observed in the crystal structure. A parallel face-to-face perfect interaction is visible in figure 1*c*, with an interplanar distance of 3.5 Å and a small shift distance of 0.3 Å. Offset or slipped packing can also be observed between the phenyl rings in the neighbouring Me_6_-SubPc molecules with an interplanar distance of 3.5 Å and a shift distance of 1.7 Å (figure 1*d*). Various weak hydrogen bonding interactions, such as between C(1)H–Cl(1) and C(1)H–N(3) as well as between C(10)H–Cl(1) and C(10)H–N(3), also contribute to the highly efficient, closely packed crystal structure, which should be favourable for carrier transport.

**Table 2.** Selected bond lengths (Å) and angles (°) for Me_6_-SubPc.

Cl(1)–B(1)	1.853(5)	N(1)–B(1)–Cl(1)	115.0(3)
N(1)–B(1)	1.497(6)	N(3)–B(1)–Cl(1)	112.8(2)
N(3)–B(1)	1.481(4)	N(1)–B(1)–N(3)	105.1(3)
N(1)–C(5)	1.369(3)	B(1)–N(1)–C(5)	122.30(18)
N(2)–C(5)	1.344(4)	B(1)–N(3)–C(6)	123.2(3)
N(2)–C(6)	1.345(4)	B(1)–N(3)–C(15)	122.9(3)
N(3)–C(6)	1.363(4)	N(1)–C(5)–N(2)	123.0(3)
N(3)–C(15)	1.370(4)	N(2)–C(6)–N(3)	122.2(3)
N(4)–C(15)	1.343(4)	N(3)–C(15)–N(4)	122.2(3)

**Figure 2.**
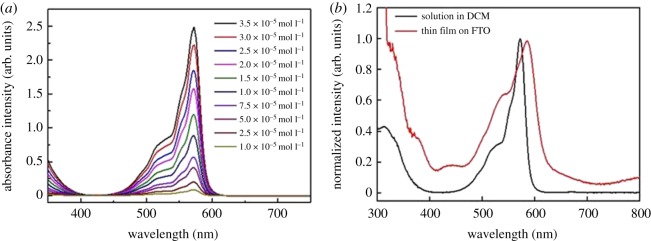
UV–Vis absorption spectra of (*a*) different concentrations of Me_6_-SubPc in DCM and (*b*) a comparison of the normalized solution spectra from panel (*a*) and a deposited film of Me_6_-SubPc on FTO.

Figure 2*a* shows the solution UV–Vis absorption spectra of different concentrations of Me_6_-SubPc. Me_6_-SubPc has a good solubility in DCM, chlorobenzene, dichlorobenzene and other organic solvents. The UV–Vis absorption spectra of a solution and film are shown in figure 2*b*. The Me_6_-SubPc solution in DCM has strong absorption peaks at 573 nm (Q-band) and 313 nm (Soret band). There is only one strong absorption peak at about 573 nm, which is attributed to the transition to an orbitally degenerate state fitted with the Faraday A term of the Me_6_-SubPc ring monomer. The Soret band is attributed to three transitions to non-degenerate states (Faraday B terms) [34]. Compared with phthalocyanine molecules, the Soret band and Q-band of Me_6_-SubPc appear at shorter wavelengths because of the smaller π-conjugation system. The differences in the absorption spectra between Me_6_-SubPc in solution and a film of SubPc were smaller than for a solution and thin film of phthalocyanine. A 13 nm red shift in conjunction with a broader absorption for each transition was observed for both the Soret band and Q-band in solution compared with for the solid-state sample. As reported in the literature, planar phthalocyanine molecules aggregate to form crystals, as evidenced by the change and widening of the Q-band absorption [37].

**Figure 3.**
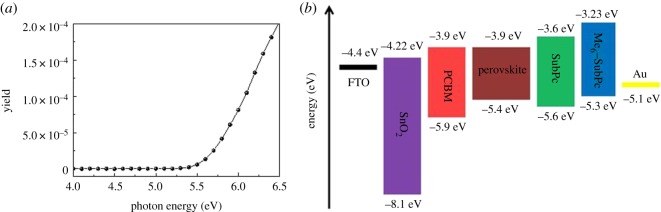
(*a*) IPS spectra of Me_6_-SubPc and (*b*) energy level diagram of the materials used in the fabricated perovskite solar cell.

The optical bandgap of Me_6_-SubPc is 2.07 eV, as estimated from the absorption edge of the solution. The HOMO level of Me_6_-SubPc was measured by IPS and was found to be 5.3 eV (figure 3*a*). The lowest unoccupied molecular orbital level of Me_6_-SubPc was calculated using the Me_6_-SubPc optical bandgap and its HOMO level; it was found to be 3.23 eV. The energy levels of the different layers of the PSCs were obtained from the literature and are shown in figure 3*b* [30,31]. As shown in figure 3*b*, the HOMO level of Me_6_-SubPc is more compatible with perovskites than the HOMO level of SubPc, and thus Me_6_-SubPc can efficiently transfer holes generated in the perovskites to the Au electrodes.

### 3.2. Solar cell performance

**Figure 4.**
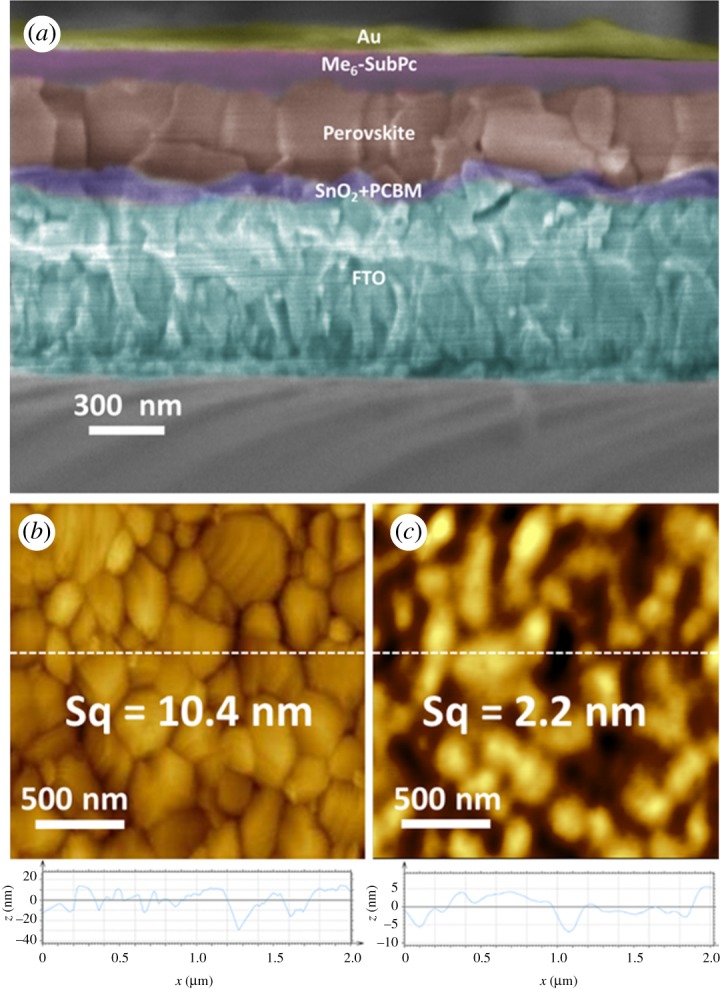
(*a*) Cross-sectional scanning electron microscopy image of FTO/SnO_2_/PCBM/perovskite/Me_6_-SubPc/Au; AFM image of (*b*) FTO/SnO_2_/PCBM/perovskite and (*c*) FTO/SnO_2_/PCBM/perovskite/Me_6_-SubPc.

The PSCs using Me_6_-SubPc as HTMs with a conventional planar device architecture consisting of FTO/SnO_2_/PCBM/perovskite/Me_6_-SubPc/Au were fabricated and characterized. Compared with TiO_2_ requiring high temperature treatment [13,49] and unstable ZnO [50,51], we chose SnO_2_ with ideal electron mobility and stability as ETL, and simultaneously used PCBM to modify the interface between perovskite and ETL to improve the electron extraction efficiency [30,31,52]. A cross-sectional scanning electron microscopy image of the device is shown in figure 4*a* and the thickness of the Me_6_-SubPc film was about 70 nm. Good contacts were formed by Me_6_-SubPc with the perovskite layer and the Au electrode. As figure 4*b*,*c* shows, the Me_6_-SubPc formed a smooth and flat film after depositing on top of the perovskite layer, which made it conducive to hole transport and hole injection.

The photovoltaic performances of PSCs using Me_6_-SubPc as HTMs were measured under a xenon lamp with air mass (AM) 1.5 solar spectrum filters and an optical power of 100 mW cm^−2^. Herein, the SnO_2_ layer (with its high electron mobility) and the effective fullerene passivation layer worked together as an electron selection layer.

**Figure 5.**
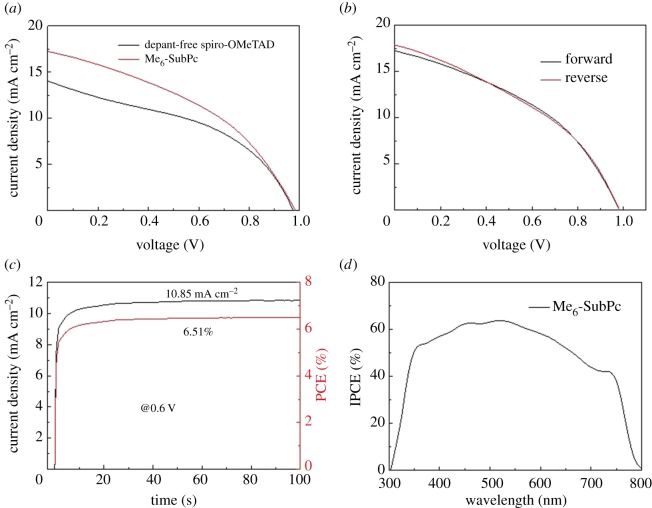
(*a*) J–V characteristics of the best devices with different HTMs and (*b*) with Me_6_-SubPc as HTM, (*c*) steady-state efficiencies of PSCs with Me_6_-SubPc as HTM at a constant bias voltage of 0.6 V. (*d*) Incident photon-to-electron conversion efficiency spectra of the best device employing the hole conductor Me_6_-SubPc.

We tested 10 PSCs in which Me_6_-SubPc was the hole transport material. Compared with previously reported results in the literature [42], devices using Me_6_-SubPc as the HTM exhibited increased *V*_oc_ and PCE values. The 10 fabricated perovskite devices with the Me_6_-SubPc HTL showed good reproducibility. The best reported device with a SubPc HTM exhibited a *V*_oc_ of 0.67 V, *J*_sc_ of 21.3 mA cm^−2^ and FF of 0.46, yielding a PCE of 6.6% [42]. The device with the Me_6_-SubPc HTL had a higher *V*_oc_ of 0.986 V, reduced *J*_sc_ of 17.21 mA cm^−2^, and FF of 0.41, with an increased PCE of 6.96%. The photovoltaic parameters of the devices with Me_6_-SubPc or dopant-free spiro-OMeTAD as their HTL are shown in table 3. As shown in figure 5*a*,*b* and table 3, the performance of the device with Me_6_-SubPc is also better than the device performance of dopant-free spiro-OMeTAD. It has been previously reported in the literature that hole transport materials with deeper HOMO levels yield PSCs with higher *V*_oc_ values [49]. In our work, the HOMO level of SubPc was −5.6 eV, which is deeper than the valence band edge and is detrimental to hole injection from the perovskites to SubPc. It is worth noting that Me_6_-SubPc has a HOMO level of −5.3 eV, which is higher than the valence band edge of perovskites and favourable for hole transport from the perovskites to Me_6_-SubPc. This reduces charge recombination at the interface and thus reduces voltage loss, which was also proved by PL spectra. As shown in the electronic supplementary material, figure S1a, there was a strong PL peak at 778 nm for the reference perovskite layer. After a spin coated Me_6_-SubPc layer on the top of perovskite, the PL spectra of perovskite films can be effectively quenched, indicating that photo-induced excitons can be separated and transferred effectively. The time-resolved photoluminescence (TRPL) spectroscopy was tested to further understand the charge transfer mechanism in PSCs with Me_6_-SubPc HTL. Electronic supplementary material, figure S1b shows the TRPL spectra of films of perovskite and perovskite/Me_6_-SubPc. It is clearly seen that the PL decay lifetime for the perovskite film is shorter than the perovskite/Me_6_-SubPc film, which also proved an efficient hole extraction from perovskite to Me_6_-SubPc. The hole mobility of Me_6_-SubPc was determined by means of hole-only devices (electronic supplementary material, figure S2) using the space charge limited current and found to be 1.4 × 10−5 cm^2^ V^−1^ s^−1^. The low hole mobilities were consistent with the reduced *J*_sc_ and FF of the devices with Me_6_-SubPc HTL. Impedance data of device was recorded and characterized by a single semicircle (electronic supplementary material, figure S3), whereby the capacitance relates to the device properties. This was also a factor affecting the *J*_sc_ and FF. Further, the J–V hysteresis of the PSCs with Me_6_-SubPc as their HTM was measured by scanning the applied voltage at 0.1 V s^−1^ in both the reverse and forward scan direction under simulated solar illumination (AM 1.5, 100 mW cm^−2^). The results are depicted in figure 5*b*, which demonstrate that the hysteresis in the Me_6_-SubPc-based devices is small. To study the stability of PSCs with Me_6_-SubPc as their HTL, a stable bias of 0.6 V was applied to the device near maximum power, and the stabilized efficiency was recorded (figure 5*c*). With Me_6_-SubPc as a HTM, the PSC achieved a stable PCE of 6.51%. To further study the performance of PSCs with Me_6_-SubPc as the HTM, the incident photon-to-electron conversion efficiencies of perovskite devices were tested to confirm the photovoltaic performance. As shown in figure 5*d*, the Me_6_-SubPc-based device exhibited enhanced IPCE over the whole visible wavelength range from 300 to 800 nm, which was in good agreement with electrical characteristics.

**Table 3.** Photovoltaic parameters of the best device for a reverse and forward scan, averaged photovoltaic parameters of 10 devices with Me_6_-SubPc as HTL and photovoltaic parameters of the best device with dopant-free spiro-OMeTAD as HTL.

	*V*_oc_ (V)	*J*_sc_ (mA cm^−2^)	FF (%)	PCE (%)
forward	0.986	17.21	41	6.96
reverse	0.986	17.88	39	6.88
average	0.954 ± 0.032	17.51 ± 0.52	39.4 ± 2.5	6.59 ± 0.38
dopant-free spiro-OMeTAD	0.974	14.02	43	5.87

## 4. Conclusion

We designed and synthesized a Me_6_-SubPc using a one-step method. Compared with unmodified subphthalocyanine, Me_6_-SubPc has a higher solubility in organic solvents, as well as a frontier orbital energy level that is well aligned with the valence band of the perovskite material. PSCs employing Me_6_-SubPc as a hole transport material were prepared using a completely solution-phase process, achieving a *V*_oc_ of 0.986 V and a PCE of 6.96%. We believe that Me_6_-SubPc molecules can be further modified such as by changing the chlorine substituent on the B central atom to help improve the as-fabricated device performance.

## Supplementary Material

Electronic Supplementary Material

## Data accessibility

Our supplementary data for the steady-state PL, time-resolved PL, experimental and calculated J–V characteristics and impedance spectroscopy characterization are available in the electronic supplementary material; the single crystal data file for hexamethyl-substituted subphthalocyanine has been deposited in Dryad and available at: http://dx.doi.org/10.5061/dryad.3d7c900 [53].
