# Supplementary material for: Soluble hexamethyl-substituted subphthalocyanine as a dopant-free hole transport material for planar perovskite solar cells
Source: R Soc Open Sci. 2018 Aug 1;5(8):180617. doi: 10.1098/rsos.180617 (PMC6124033; doi:10.1098/rsos.180617)
Supplement: Electronic Supplementary Material [file rsos180617supp1.doc]

**Electronic Supplementary Material**

Soluble hexamethyl-substituted subphthalocyanine as a dopant-free hole transport material for planar perovskite solar cells

Feng Wang, Xiaoyuan Liu, Ehsan Rezaee, Haiquan Shan, Yuxia Zhou, Zong-Xiang Xu*

Department of Chemistry, South University of Science and Technology of China, 518055, Shenzhen, Guangdong, China. Email: [xu.zx@sustc.edu.cn](mailto:xu.zx@sustc.edu.cn)


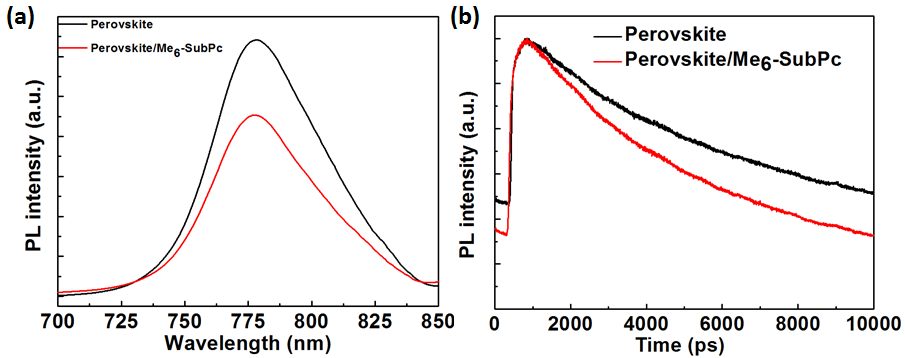


**Figure S1.** (a) Steady-state photoluminescence (PL) spectra of the bare perovskite and perovskite/Me_6_-SubPc. (b) Normalized time-resolved photoluminescence spectra of corresponding samples.


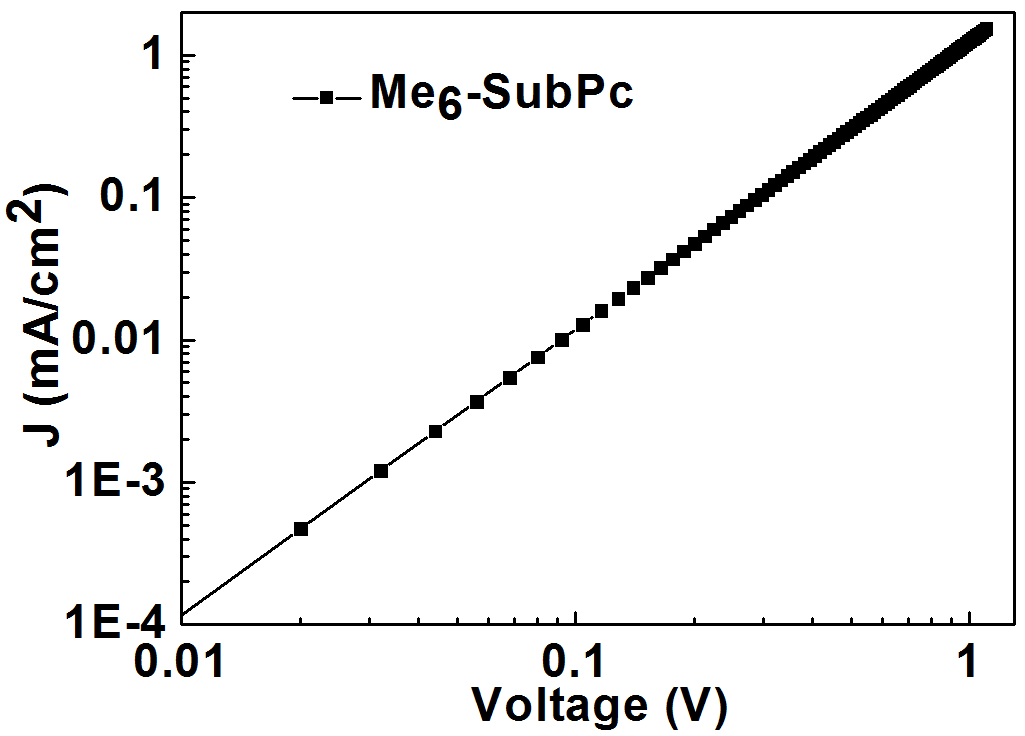


Figure S2. Experimental and calculated (solid lines) *J*–*V* characteristics of ITO/PEDOT:PSS/Me_6_-SubPc/Al devices. The device was prepared using the version of a previously reported method.[^1^](#_ENREF_1)


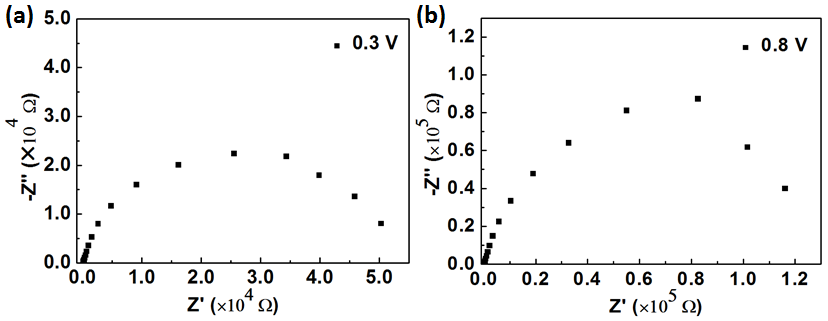


Figure S3. Impedance spectroscopy characterization with different DC bias potentials of (a) 0.3V and (b) 0.8 V
